# Surface Pattern over a Thick Silica Film to Realize Passive Radiative Cooling

**DOI:** 10.3390/ma14102637

**Published:** 2021-05-18

**Authors:** Yuhong Liu, Jing Li, Chang Liu

**Affiliations:** 1 Hebei Key Laboratory of Electromagnetic Environmental Effects and Information Processing, Shijiazhuang Tiedao University, Shijiazhuang 050043, China; liuyumin@bupt.edu.cn; 2 State Key Laboratory of Information Photonics and Optical Communications, Beijing University of Posts and Telecommunications, Beijing 100876, China; 3 State Key Laboratory of Superlattices and Microstructures, Institute of Semiconductors, Chinese Academy of Sciences, Beijing 100083, China

**Keywords:** passive radiative cooling, silica film, surface mode

## Abstract

Passive radiative cooling, which cools an item without any electrical input, has drawn much attention in recent years. In many radiative coolers, silica is widely used due to its high emissivity in the mid-infrared region. However, the performance of a bare silica film is poor due to the occurrence of an emitting dip (about 30% emissivity) in the atmospheric transparent window (8–13 μm). In this work, we demonstrate that the emissivity of silica film can be improved by sculpturing structures on its surface. According to our simulation, over 90% emissivity can be achieved at 8–13 μm when periodical silica deep grating is applied on a plane silica film. With the high emissivity at the atmospheric transparent window and the extremely low absorption in the solar spectrum, the structure has excellent cooling performance (about 100 W/m^2^). The enhancement is because of the coupling between the incident light with the surface modes. Compared with most present radiative coolers, the proposed cooler is much easier to be fabricated. However, 1-D gratings are sensitive to incident polarization, which leads to a degradation in cooling performance. To solve this problem, we further propose another radiative cooler based on a silica cylinder array. The new cooler’s insensitivity to polarization angle and its average emissivity in the atmospheric transparent window is about 98%. Near-unit emissivity and their simple structures enable the two coolers to be applied in real cooling systems.

## 1. Introduction

**Figure 1:**
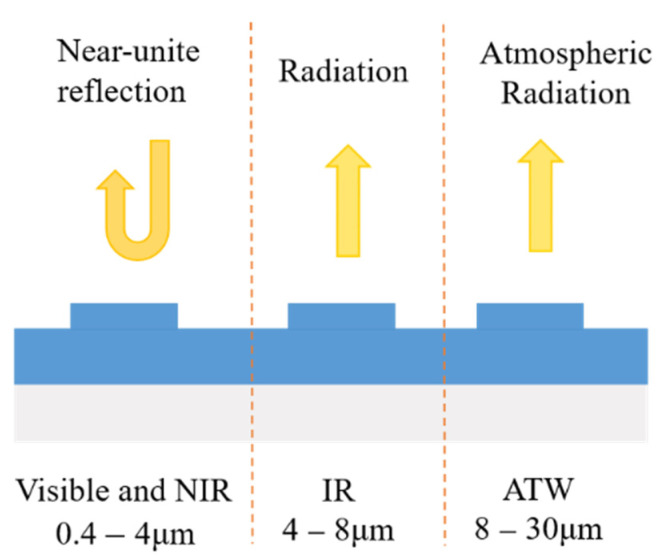
Scheme of solar reflection and the thermal radiation properties of a daytime radiative cooler. ATW refers to the atmospheric transparent window [14].

Radiative cooling is necessary in many semiconductor devices where undesired heating takes place. The heat arising within devices, together with the heat from the operating atmosphere, results in a decrease in working efficiency and reliability. For example, a 1 °C temperature increase leads to an approximately 0.45% efficiency decrease in a crystalline silica solar cell [1]. In addition, the aging rate of a solar cell array doubles the increase in the solar cell temperature [2]. Therefore, cooling techniques have a crucial role in maintaining operating temperatures within proper ranges. Instead of conventional active cooling techniques, such as forced air flow [3], heat-pipe-based systems [4], and water cooling [5], passive radiative cooling can achieve a cooling effect of objects below ambient temperatures, without any secondary energy consumption [6], such as electric energy, gasoline, or diesel oil [6]. The main concept of designing a passive radiative cooler is tailoring the spectral emission and spectral absorption of an optical structure. The transparency window for electromagnetic waves of the Earth’s atmosphere is between 8–13 μm, which coincides with the peak thermal radiation wavelength at typical ambient temperatures. Therefore, one can cool down a body on the Earth’s surface by radiating its heat away and into cold outer space through this spectral window. Radiative coolers can be divided into daytime and nighttime coolers, according to their operating times. For nighttime radiative coolers, the spectral emission should be as high as possible in the 8–13 μm wavelength range. A great deal of research has been done in this field in the past few decades [7,8,9,10,11,12,13]. In addition to the high emission of 8–13 μm, a daytime cooling design also requires near unit reflectivity in the visible and near-infrared (NIR) spectrum to effectively reflect solar light, as shown in Figure 1. For daytime radiative coolers, the absorption of the solar spectrum should be as low as possible. An ideal radiative cooler requires zero absorption in the solar spectrum and the unit emissivity should be in the atmospheric window. Although there are many broadband absorbers and reflectors that have been proposed [14,15,16,17,18,19,20], the design of a radiative cooler is more difficult since it requires a high absorption and high reflection at specific wavelengths [21].

**Figure 2:**
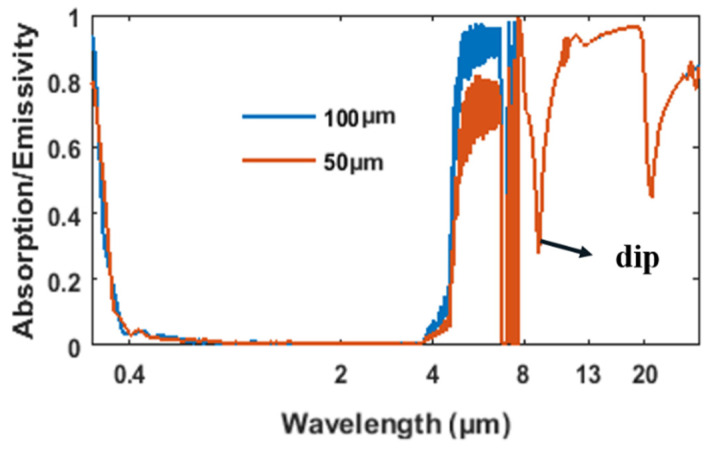
Absorption/emissivity of pure silica films with different thicknesses placed over a sliver substrate, calculated using the transfer matrix method [30].

Previous works on daytime radiative cooling have shown remarkable achievements [22,23,24,25,26,27,28,29]. For example, Raman, Li, and Ali proposed high-index and low-index alternating layers with different materials and thicknesses to realize radiative cooling in their respective works [22,24,25]. A surface patterned multi-layer photonics crystal structure was proposed by Rephaeli to realize radiative cooling [23]. Zhu proved that tapered photonics crystals can realize near-ideal emittance in the transparent window and also achieved a high radiative cooling efficiency in their work [26]. Most of these efficient radiative cooling structures applied silica as one of the main materials in their designs due to its relatively high emissivity in the 8–13 μm range, which coincides with the atmosphere transparent window, and also has an extremely low absorption at 0–4 μm. However, the emissivity of a pure bulk silica exhibits a large dip near 9 μm due to the bulk phonon–polariton excitation of silica [24]. This dip coincides with the transparency window of the atmosphere and ultimately influences the efficiency of a radiative cooler. As shown in Figure 2, the existence of the emitting dip greatly degrades the overall emissivity in the transparent window. Therefore, it is important to improve the emissivity around 9 μm by degrading the bulk phono–polariton effect for silica-based radiators.

Solutions have been proposed to deal with this problem in previous works. One such method is to apply new materials to improve emissivity in a structure. Metallic oxides, such as alumina, titania, as well as some other metallic compounds, can increase the emissivity of silica due to their good emissivity at around 9 μm. With this in mind, alternating dielectric layer structures has been proposed in [22,24,25] and the emissivities in these structures has been proven to be over 80% at around 9 μm. However, a weakness of this method is that the introductions of other materials into a pure silica structure may cause undesired absorption in the visible and near-infrared region. Meanwhile, it is difficult for the emissivity of these structures to reach 90% or higher in the entire transparent window due to the influence of other materials. Another way to decrease the effect of bulk silica phonon–polariton is surface tailoring. By carving the planar surface of a pure silica film into micro- or nano-silica structures, the emission at around 9 μm can be partially promoted. Using this method, the emissive ability of silica in the transparent window can often be maximized. However, the micro- or nano-silica structures present in these types of radiative coolers might be too complicated to fabricate on a large scale. This weakness makes them difficult to be applied practically. Therefore, there is still much to be done to design highly efficient radiative coolers with simple structures.

In this paper, we propose and theoretically demonstrate that simple micro-structures, such as 1-D silica grating, can improve emissivity in the atmospheric transparent window and realize radiative cooling. As one-dimensional gratings are the simplest patterned photonic crystal structures, their fabrications are much easier since only a single polarization operation is necessary [31]. We will discuss how grating improves the emissivity of the structure. Further, we will improve our structure to overcome the shortfall of a very poor polarization sensitivity and introduce a cylinder-array structure. Finally, we will evaluate the cooling performances of these coolers. All the proposed cooling structures in our paper are simple and their sizes are all in the micron range. These advantages make them much easier to be fabricated on a large scale.

## 2. Physical Model

**Figure 3:**
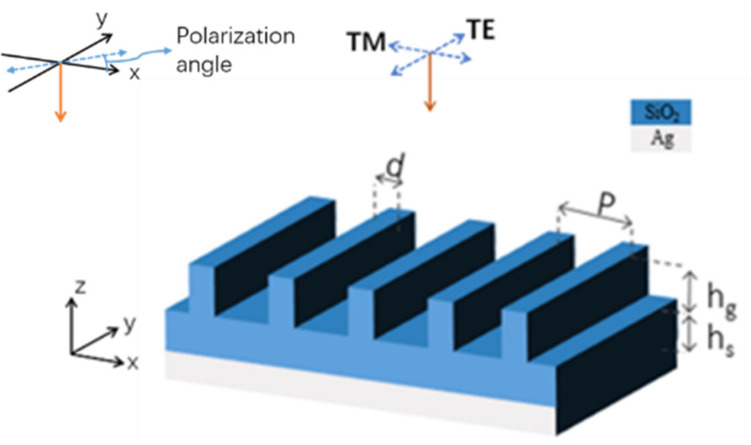
Basic structure of proposed silica grating. The period (*P*) of the grating is 12 μm. The ridge width (*d*) of the grating is 2 μm. The thickness of the silica layer (*h_s_*) is 100 μm. The height (*h_g_*) of the grating is 18 μm. The height (*h_g_*) can be reduced to 6 μm by sacrificing the absorption efficiency. The blue dotted line with double arrows represents the direction of the incident electric field.

Our basic structure is depicted in Figure 3. A silica grating lies over a thick silica layer and beneath the silica layer is a silver substrate functioning as a reflective mirror. The period of the grating was set as 12 μm, which is close to the spectral wavelength, ranging from 8 to 13 μm, where the emissivity needs to be improved. The silica layer beneath the grating structure had a thickness of 100 μm (marked as “*h_s_*” in Figure 3), which should be thick enough to realize high emissions in the transparent window. As for substrate, silver was preferred in our structure because it barely absorbs light in the visible and near-infrared regions. We should also note that some other metals, such as tungsten, platinum and iron, are not suitable to be applied as the metal substrate due to their relatively high absorption in the visible and near-infrared (NIR) regions, resulting from their moderate extinction coefficients [32]. Undesired heat can be induced from these metal substrates from their absorption of solar energy, which eventually degrades the cooling efficiency.

For the simulations, the 2-D finite difference time domain (FDTD) method was performed in this case. Due to the complex situations of the ultra-broad operating waveband, we simulated the structure with various mesh sizes, at different wavelength ranges (0–2, 2–4, 4–8, 8–10,10–20, and 20–30 μm), ensuring the accuracy of the simulation result. The refractive index of the materials used in this paper adopted experimental data [32,33]. Periodical boundary conditions were applied on both sides of a simulated cell. Sufficient perfectly matched layers (PMLs) are imposed on both the top and the bottom of the structure. A transverse-magnetic (TM) light wave was normally incident along the negative z-direction with the polarization along the x-direction. The absorption of the structure was calculated as *A*= 1 − *R* (reflection), owing to the opaque silver substrate (*T* = 0). The emissivity of the structure could be represented as ε = *A* = 1 − *R* according Kirchhoff’s law.

## 3. Results and Discussions

**Figure 4:**
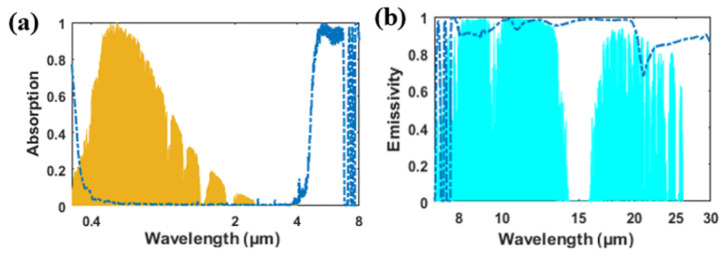
(**a**) Absorption of grating structure in Figure 1; normalized AM 1.5 solar spectrum is plotted in orange. (**b**) Emissivity of grating structure in Figure 1 in the atmosphere transparent window. The atmospheric transmittance is plotted in blue.

Figure 4 shows the absorption/emissivity of the structure. In the solar spectrum (about 0.3 to 4 μm), the absorption of the structure was extremely low, indicating that the structure barely absorbed solar light. The average absorption in the solar spectrum can be calculated as:

(1) $$\bar{A}=\int_{\lambda_{1}}^{\lambda_{2}}A(\lambda )d\lambda /(\lambda_{2}-\lambda_{1})$$

According to the calculation, the average absorption for the structure in the solar spectrum (0.3–3.7 μm) was about 2.2%. With little loss, solar light penetrated the silica structure and was almost reflected by the opaque metal substrate. In Figure 4b, the emissivity of the grating structure exceeds 90% in the entire atmospheric transparent window. With the adoption of the grating, there is no obvious emitting dip appearing in the atmospheric transparent window (8 to 13 μm) for the grating structure.

**Figure 5:**
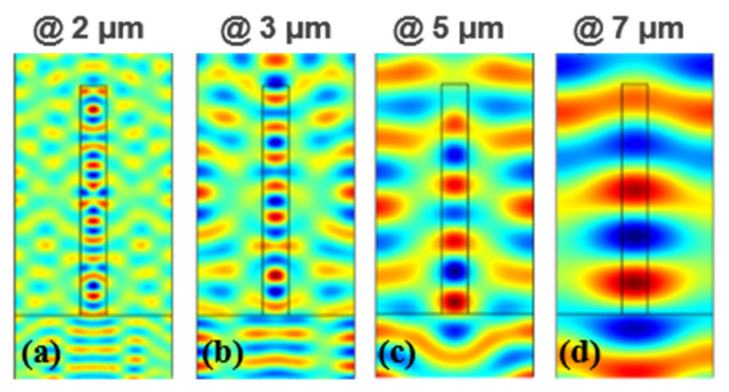
Spatial distribution of the magnetic field (*H_y_*) at the wavelength of (**a**) 2μm, (**b**) 3 μm, (**c**) 5 μm, (**d**) 7 μm. Red and blue represent the maximum and minimum magnitudes, respectively.

The emissivity enhancement in the grating structure was mainly caused by the coupling of incident light to the in-plane grating mode. When TM polarized the wave incident normally to the grating, multiple longitudinal guided modes arose and propagated along the y direction. To gain a deeper insight into these modes, the spatial distributions of the magnetic field (*H_y_*) of the grating at different wavelengths were calculated. For simplicity, we divided the whole spectrum into three parts, namely 0.3–3.7, 3.7–8, and 8–30 μm, according to the differences of these modes. In Figure 5a,b, the internal field distribution in the shorter wavelength range (0.3–3.7 μm) shows a pattern akin to a TM1 slab waveguide mode. The guided modes appearing in the grating also induce the occurrence of guided modes in the silica layer beneath the grating. In lossy dielectric structures, these guided modes will result in an increase in the absorption of incident light. However, the extinction coefficient (*k*) of silica is zero in the 0.3–3.7 μm wavelength range. Therefore, light penetrates the silica grating without any loss. The occurrence of these modes will not increase the absorption in this region. The absorption is mainly caused by the silver substrate. In Figure 5a, the absorption of the grating and the absorption of the structure without the grating in the solar spectrum are plotted for comparison. The absorption of the grating structure is almost the same as the absorption of structure without the grating (shown in Figure 5a), proving that the grating cannot increase the absorption in the solar spectrum.

**Figure 6:**
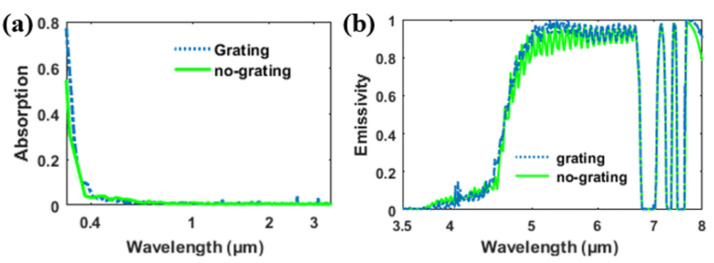
Absorption/emissivity for the proposed structure with/without the grating at (**a**) 0–3.7 μm and (**b**) 3.5–8 μm.

When the wavelength of the incident light is increased but is less than 7 μm, the extinction coefficient (*k*) of silica is silica is no longer transparent, but its absorption loss is low (the extinction coefficient is less than 0.02) [31]. In many optical systems, the loss of materials with extinction coefficient less than 0.02 can be neglected. However, the thickness of the silica layer *(h_s_*) is very large in our structure (100 μm), thus, the extremely small extinction coefficient cannot be ignored. In Figure 5c,d, the magnetic fields at different wavelengths in this region are plotted. The longitude guided modes still exist in the grating and the silica substrate at these wavelengths. The absorption/emissivity of the grating structure and the no-grating structure in the range of 3.5–8 μm are plotted in Figure 6b. Unlike the absorption spectrum in the solar wavelength, the absorption here shows a near-unit absorption in some wavebands. Additionally, the two absorption spectra almost coincide. These results indicate that the high absorption/emissivity in the region 4–7 μm is mainly caused by the thick dielectric layer, while the grating only leads to a small increase in the absorption. We should also note that the absorption at around 7–8 μm is zero in several discontinuous wavelength ranges. This is due to the imaginary part of the silica reflective index being zero within these discontinuous wavelength ranges according to the data we applied [34]. Therefore, light penetrates the structure without any loss and is totally reflected by the silver substrate.

**Figure 7:**
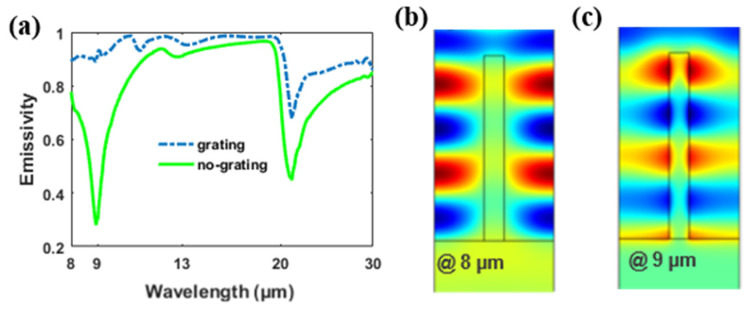
(**a**)Absorption/emissivity for the proposed structure with or without the grating at 8–30 μm. (**b**,**c**) Spatial distribution of magnetic field (*H_y_*) at different wavelengths in the grating structure. Red and blue represent the maximum and minimum magnitudes, respectively.

When the wavelength is longer (near 8 μm), the extinction coefficient (*k*) of silica increases while the refractive index (*n*) of silica drops rapidly to below one. The refractive index of silica is about 0.41 + 0.32*i* at λ = 8 μm [34], indicating that silica functions as a lossy dielectric at this case. The magnetic field distribution at λ = 8 μm is plotted in Figure 7b. Instead being confined in the grating, a standing wave pattern is clearly visible in the middle of the air grooves. In addition to this, there is no field pattern appearing in the silica layer beneath the substrate. To better understand the occurrence of the standing wave in the air groove, we consider an open Fabry-Perot (FP) cavity, which has two mirrors of finite reflectivity at both ends of the air groove. When light is normally incident to the cavity, one part of the light is reflected by the silica substrate and the other part of the light penetrates the silica substrate and dampens rapidly due to the high losses of the silica. The guided mode is reflected at the open end of the air groove, so that a standing wave pattern occurs due to the coupling of the incident and reflected light. The resonance wavelength of the Fabry-Perot resonance under open-cavity conditions can be determined by [35,36,37,38,39]:

(2) $$\frac{2m}{4}\lambda =n_{eff}h_{g}$$

where $h_{g}=18\;\mu m$ is the height of the grating, $n_{eff}$ is the effective refractive index of the grating, *m* is the positive integer and counts the number of the sum of the field maximum and field minimum of the standing wave pattern of magnetic field *H_y_*. At TM incidence, $n_{eff}^{TM}=\varepsilon_{⊥}^{1/2}={\left[1+\left(\varepsilon_{\mathrm{SiO}2}^{-1}-1\right)f\right]}^{-0.5}$, where f is the filling factor of the grating. At λ=8 μm, $n_{eff}^{TM}=0.8978+0.2515i$. Ignoring the imaginary part of $n_{eff}^{TM}$, the right side of Equation (2) is 16.16, which approximately equals with the left side of Equation (2) (about 16) at m=4. The validity of the equation indicates the existence of F-P resonance within the structure. In Figure 7a, we plot the emissivity of the grating structure and the no-grating structure. Apparently, there is an increase in the emissive efficiency, which results from the F-P resonance in the grating.

When the wavelength of the incident light is near 9 μm, another distinctive standing wave pattern occurs, as plotted in Figure 7c. The standing wave is confined around the grating instead of the air groove, which means that another standing wave mode occurs. The corresponding refractive index of silica at 9 μm is about 0.58 + 2.27*i*. Instead of functioning as a high-loss dielectric, the grating shows a metallic property, because its permittivity is −4.8165 + 2.6332*i*. Therefore, most of the incident light is reflected by the thick silica film and the cavity can no longer be regarded as an open cavity. Instead, it can be considered a closed cavity. For a closed cavity, the resonance condition changes into:

(3) $$\frac{2m-1}{4}\lambda =n_{eff}h_{g}$$

At λ=9 μm, $n_{eff}^{TM}=1.1135+0.0102i$. Ignoring the imaginary part of $n_{eff}^{TM}$, the right side of Equation (3) is 20.04, which approximately equals the left side of Equation (3) (about 20.25) at m=5. The validity of Equation (3) proves the resonance to be a closed cavity resonance. Shown in Figure 7a, the resonance gives rise to a large increase in the emissivity of the structure. The emissive dip for the silica layer can be well inhibited through cavity resonance at around 9 μm. The emissivity enhancement in the atmosphere of 8–13 μm proves that the grating is efficient in this structure.

For a longer wavelength range (13–20 μm), the emissivity of bare thick silica film (green solid line) is already high enough. Therefore, the emitting enhancement resulting from the silica grating is not so obvious as it with 8–9 μm. However, the emitting enhancement caused by the grating exists at this wavelength range and is shown in Figure 7a.

**Figure 8:**
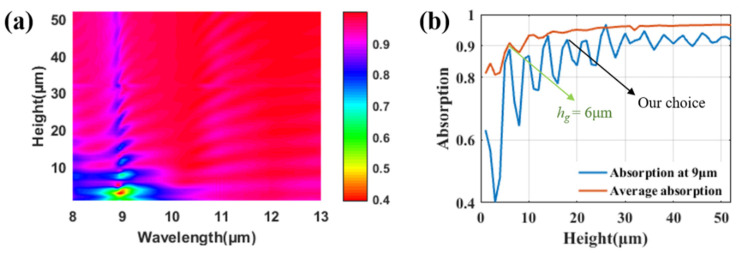
(**a**) Emissivity of the proposed structure varying with the height of the grating (*h_g_*) in the atmospheric transparent window. (**b**) Blue line: emissivity at 9 μm varying with the height of the grating. Red line: average absorption in the atmospheric transparent window.

The height of the grating is crucial to reach different orders of cavity resonances. As shown in Figure 7c, there are various longitudinal guided modes that arise within or around the grating ridge at around 9 μm. For the purpose of improving the absorption or emissivity of the structure, the incident light should be well consumed by the grating structure. Therefore, the number of longitudinal modes should be sufficient to well confine the incident light and the height of the grating should be tall enough to support these high-order modes. Generally speaking, the number of longitudinal modes will increase with the increase in the height (*h_g_*), resulting in an increase in the emissivity of the structure. However, this is partly true in our case. To better illustrate the relationship between the height (*h_g_*) with the emissivity of the structure, we numerically calculate this result in Figure 8. In Figure 8a, the emissivity of the structure grows with the increase in the height of the grating, as we expected. However, there are emissivity dips that appear periodically with the increase in height (*h_g_*), indicating that the height of the grating performs a very important role in the emissivity. To gain further insight into this phenomenon, we exclude the emissivity at 9 μm in Figure 8b. The emissivity at 9 μm increases with the increase in hg with alternating dips and peaks appearing. This can be easily understood by explaining that the cavity resonance condition is valid at every height of the emitting peak in Figure 8b, which gives rise to the emissivity enhancement of the structure, while the emissivity enhancement is not so prominent at every emitting dip in Figure 8b where the cavity resonance is no longer valid. With the increase of height (*h_g_*), the higher ordered modes appear within the grating and the emissivity improves at every emitting peak. The emissivity of the structure reaches its limit when *h_g_* is over 30 μm at 9 μm. The average emissivity (plotted as a red line in Figure 8b) reaches over 90% when the height is 6 μm. We choose *h_g_* as 18 μm in our paper, because the emissivity reaches over 90% in the whole atmospheric transparent window. The high-ordered cavity resonances are more easily observed when the height is large. Indeed, considering the absorption efficiency and producing difficulty, a height of 6 μm is enough for the structure.

**Figure 9:**
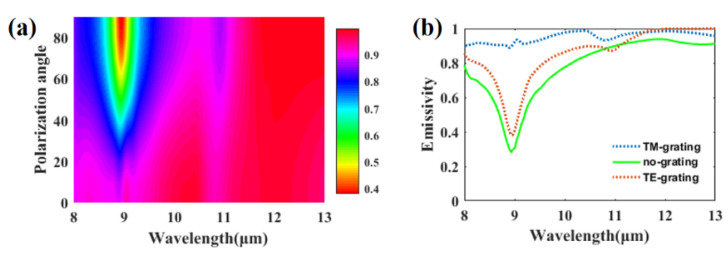
(**a**) Emissivity of the proposed structure at atmospheric transparent window varying with polarization angle. (**b**) Emissivity of the proposed structure at TM and TE incidence.

For 1-D photonic grating, its optical response is often sensitive to polarization because of its high anisotropy property. We plot the emissivity of the structure in the atmosphere transparent window, varying with polarization angle, in Figure 9a. Obviously, the emissivity at around 9 μm decreases rapidly with the increase in the polarization angle. This phenomenon can be illustrated by the anisotropy of the average refractive index. Using zero-order effective medium approximation [40], the effective refractive index of the grating at TM and transverse-magnetic (TE) polarization are given as:

(4) neffTM=[εPcosθsinφ+ε^(1−cos2θsin2φ)1/2]1/2

(5) neffTM=[εPcosφ+ε^sinφ]1/2

where $\varepsilon_{∥}=1+\left(\varepsilon_{\mathrm{SiO}2}-1\right)f$ and $\varepsilon_{⊥}={\left[1+\left(\varepsilon_{\mathrm{SiO}2}^{-1}-1\right)f\right]}^{-1}$ with f=d/P denoting the filling factor of the grating and $\varepsilon_{\mathrm{SiO}2}$ is the dielectric constant of silica. At normal incident where φ=0, Equations (1) and (2) can be simplified as $n_{eff}^{TM}=\varepsilon_{⊥}^{1/2}$ and $n_{eff}^{TE}=\varepsilon_{∥}^{1/2}$. At incident wavelength λ=9 μm, $n_{eff}^{TM}=1.1135+0.0102i$, which is close to 1 according to our calculation, while $n_{eff}^{TE}=0.4889+0.4420$. This indicates that the grating functions as an antireflection film at TM incidence because the efficient refractive index is close to the refractive index of free space. At TE incidence, $real(n_{eff}^{TE})=0.4889$, which is close to the refractive index of silica $real\left(n_{SiO2}\right)=0.5757$. Meanwhile, the extinction coefficient of the effective refractive index of the grating (0.44) and the silica (2.25) are very large. With a low refractive index and high extinction coefficient, the optical response of the grating and the silica layer are relatively similar at the TE incident. Therefore, the grating function, as an extra silica layer and the emissivity of the grating structure at the TE incidence, is relatively similar with the emissivity of the planar structure without grating (plotted in Figure 9b).

**Figure 10:**
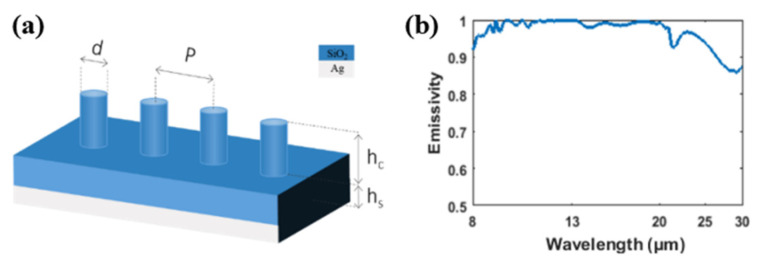
(**a**) Improved structure: patch-antenna-like cylinder array. The period (*P*) of the cylinder is 12 μm. The diameter (*d*) of the cylinder is 4 μm. The thickness of the silica layer (*h_s_*) is 100 μm. The height (*h_c_*) of the cylinder is 30 μm. (**b**) Emissivity of the proposed structure in the atmospheric transparent window.

Thus far, we have proved that radiative cooling can be realized in 1-D silica gratings. However, the polarization insensitivity of this radiative cooler is poor due to the high anisotropy of the structure. Building on the basic grating structure, we propose another radiative cooling structure that has a rotational symmetry property; hence, it is insensitive to polarization. The structure is plotted in Figure 10a. Instead of grating, we place a cylinder array over a thick silica film and propose a 3-D patch-antenna-like structure. The emissivity of the structure in the transparent window is plotted in Figure 10b. Obviously, the emissivity of the structure is close to 9 μm to around 20 μm, indicating its excellent radiative cooling performance. Without applying new materials into the structure, the absorption in the solar spectrum remains similar to the absorption of grating structure with an average absorption of around 2.2%, from 0.3 to 3.7 μm. The emissivity of the cylinder structure is relatively better than the grating structure due to its greater height which can support higher-order modes.

In addition to the influence of the emission profile of the cooler, the cooling efficiency of a radiative cooler can be influenced by many factors, including the nonradiative heat obtained from the surrounding media. For a cooling structure, the net cooling power (*P_cool_*) of the cooler is defined as:

(6) $$P_{cool}=P_{rad}-P_{ATM}-P_{Sun}$$

where $P_{Rad}$ is the power emitted by the proposed radiative cooler, $P_{Atm}$ is the absorbed power from the incident atmospheric radiation, and $P_{Sun}$ is the power absorbed from the sun. They can be respectively calculated by:

(7) $$P_{rad}(T)=A\int_{0}^{2\pi }d\theta \cos\theta \int_{0}^{\infty }d\lambda I_{BB}(T,\lambda )\varepsilon (\lambda ,\theta )$$

(8) $$P_{ATM}(T_{ATM})=A\int_{0}^{2\pi }d\theta \cos\theta \int_{0}^{\infty }d\lambda I_{BB}(T_{ATM},{\lambda )\varepsilon }_{\mathrm{ATM}}(\lambda ,\theta )$$

(9) $$P_{Sun}(T)=A\int_{0}^{\infty }d\lambda {\varepsilon (\lambda ,\theta }_{\mathrm{Sun}})I_{AM1.5}(\lambda )$$

where $I_{BB}\left(T,\lambda \right)=2hc^{2}/\lambda^{5}\left(e^{\frac{hc}{\lambda kT}}-1\right)$ is the spectral radiation intensity of a blackbody according to Planck’s law. T is the operating temperature, c is the speed of light, h is the Planck’s constant, and k is Boltzmann’s constant. In Equation (7), ε(λ,θ) is the directional emissivity of the radiative cooler at wavelength λ, which is equal to its absorptivity according to Kirchoff’s law. In Equation (8), the angle dependent emissivity of the atmosphere can be calculated by ε_ATM_(λ,θ) = 1 − *t*(λ)1/cosθ at the wavelength of λ, where *t*(λ) is the atmospheric transmittance in the zenith direction. Equation (9) is the solar absorption of the radiative cooler.

**Figure 11:**
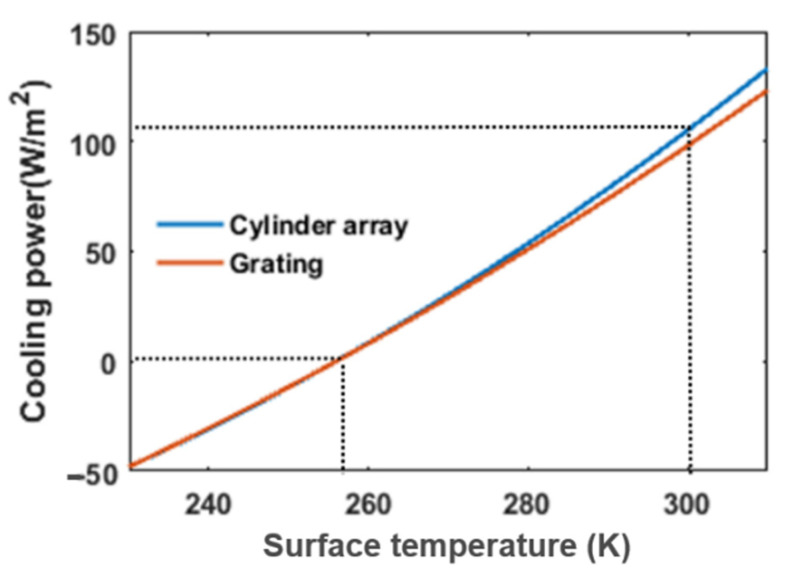
Cooling performance of proposed structures.

As we calculate in Figure 11, the net cooling power of the grating cooler is about 98 W/m^2^ while the net cooling power of the cylinder cooler is about 105 W/m^2^. At room temperature, both of the coolers have a cooling efficiency of around 100 W/m^2^. For these two radiative coolers, their absorption in the solar spectrum is extremely low because the extinction coefficient of silica is zero in the solar spectrum from 0.3 to about 3.7 μm. Their emissivities can reach over 90% in the entire atmospheric transparent window. Therefore, their cooling performances are similar.

## 4. Conclusions

In conclusion, we numerically demonstrate a high-performance radiative cooler based on silica grating. The structure has good cooling performance, resulting from the high emissivity in the atmosphere transparent window and an extremely low absorption in the solar spectrum. The cavity modes that arise within the structure surface greatly enhance the emissivity in the atmospheric transparent window. To solve the shortcomings of the polarization sensitivity, we further propose a cylinder array structure, which is insensitive to the polarization due to its rotational symmetry. According to our calculations, the cooling efficiencies of these two structures are around 100 W/m^2^. The structures of these two coolers are simple, which allow wide fabrication and applications in real heat-generated systems.

## Data Availability

Data sharing is not applicable to this article.
